# A Phase 3, Randomized, Double-Blind Study Comparing Tedizolid Phosphate and Linezolid for Treatment of Ventilated Gram-Positive Hospital-Acquired or Ventilator-Associated Bacterial Pneumonia

**DOI:** 10.1093/cid/ciab032

**Published:** 2021-03-15

**Authors:** Richard G Wunderink, Antoine Roquilly, Martin Croce, Daniel Rodriguez Gonzalez, Satoshi Fujimi, Joan R Butterton, Natasha Broyde, Myra W Popejoy, Jason Y Kim, Carisa De Anda

**Affiliations:** 1 Department of Medicine, Division of Pulmonary and Critical Care, Northwestern University Feinberg School of Medicine, Chicago, Illinois, USA; 2 Université de Nantes, Centre Hospitalier Universitaire de Nantes, EA3826 Thérapeutiques Anti-Infectieuses, Service d’Anesthésie Réanimation Chirurgicale, Hôtel Dieu, Nantes, F-44000; 3 Regional One Health, Memphis, Tennessee, USA; 4 Department of Intensive Care, Nuevo Hospital Civil de Guadalajara, Guadalajara, Jalisco, Mexico; 5 Department of Trauma, Critical Care, and Emergency Medicine, Osaka General Medical Center, Sumiyoshi-ku, Osaka, Japan; 6 Merck Research Laboratories, Merck & Co, Inc, Kenilworth, New Jersey, USA

**Keywords:** gram-positive cocci, healthcare-associated bacterial pneumonia, Staphylococcal infections, ventilator-associated bacterial pneumonia

## Abstract

**Background:**

Hospital-acquired bacterial pneumonia (HABP) and ventilator-associated bacterial pneumonia (VABP) are associated with high mortality rates. We evaluated the efficacy and safety of tedizolid (administered as tedizolid phosphate) for treatment of gram-positive ventilated HABP/VABP.

**Methods:**

In this randomized, noninferiority, double-blind, double-dummy, global phase 3 trial, patients were randomized 1:1 to receive intravenous tedizolid phosphate 200 mg once daily for 7 days or intravenous linezolid 600 mg every 12 hours for 10 days. Treatment was 14 days in patients with concurrent gram-positive bacteremia. The primary efficacy end points were day 28 all-cause mortality (ACM; noninferiority margin, 10%) and investigator-assessed clinical response at test of cure (TOC; noninferiority margin, 12.5%) in the intention-to-treat population.

**Results:**

Overall, 726 patients were randomized (tedizolid, n = 366; linezolid, n = 360). Baseline characteristics, including incidence of methicillin-resistant *Staphylococcus aureus* (31.3% overall), were well balanced. Tedizolid was noninferior to linezolid for day 28 ACM rate: 28.1% and 26.4%, respectively (difference, –1.8%; 95% confidence interval [CI]: –8.2 to 4.7). Noninferiority of tedizolid was not demonstrated for investigator-assessed clinical cure at TOC (tedizolid, 56.3% vs linezolid, 63.9%; difference, –7.6%; 97.5% CI: –15.7 to 0.5). In post hoc analyses, no single factor accounted for the difference in clinical response between treatment groups. Drug-related adverse events occurred in 8.1% and 11.9% of patients who received tedizolid and linezolid, respectively.

**Conclusions:**

Tedizolid was noninferior to linezolid for day 28 ACM in the treatment of gram-positive ventilated HABP/VABP. Noninferiority of tedizolid for investigator-assessed clinical response at TOC was not demonstrated. Both drugs were well tolerated.

**Clinical Trials Registration:**

NCT02019420.

## Teaser

Tedizolid was noninferior to linezolid for all-cause mortality in the treatment of ventilated gram-positive hospital-acquired or ventilator-associated bacterial pneumonia. Noninferiority of tedizolid to linezolid for investigator-assessed clinical response at test of cure was not demonstrated. Both drugs were well tolerated.

Hospital-acquired bacterial pneumonia (HABP) and ventilator-associated bacterial pneumonia (VABP) are among the most common healthcare-associated infections [1–4]. Estimated global all-cause mortality (ACM) for HABP/VABP is 10% to 39% and attributable mortality is 4% to 13% [5–8]. Globally, *Staphylococcus aureus* is one of the most commonly identified causative gram-positive pathogens in HABP/VABP, with associated mortality rates of approximately 30% to 40% [1, 2, 9, 10].

Tedizolid phosphate is an oxazolidinone prodrug that endogenous phosphatases convert in vivo to the active moiety tedizolid, which inhibits bacterial protein synthesis [11]. In vitro, tedizolid has broad activity against gram-positive pathogens, including methicillin-, vancomycin-, and certain linezolid-resistant strains of *S. aureus* [11–14]; in vitro potency of tedizolid was 4- to 8-fold greater than linezolid across a range of gram-positive pathogens [13–15]. From 2009 through 2013, the Surveillance of Tedizolid Activity and Resistance program tested >11 000 gram-positive clinical isolates from the United States and Europe, including respiratory tract specimens, and found that tedizolid inhibited 99.7% of isolates at a minimum inhibitory concentration (MIC) of ≤0.5 mg/L [16].

Tedizolid phosphate is approved for the treatment of acute bacterial skin and skin structure infections (ABSSSIs) as an oral or intravenous (IV) 200-mg dose administered once daily for 6 days [11, 17, 18]. Tedizolid demonstrates excellent pulmonary penetration in healthy adult participants, with epithelial lining fluid (ELF) concentrations higher than free plasma concentrations for the entire dosing interval and an approximately 40-fold ELF-to-plasma penetration ratio [19].

In our trial, we compared a 7-day course of tedizolid phosphate with a 10-day linezolid course for treatment of ventilated HABP/VABP. The 7-day tedizolid phosphate duration was chosen based on previous trials that demonstrated no differences in outcomes with short (7–8 days) vs long treatment courses (10–15 days), as well as current Infectious Diseases Society of America/American Thoracic Society clinical practice guidelines for HABP/VABP [20–22]. Consistent with US Food and Drug Administration (FDA) guidance for HABP/VABP clinical trials, a 10-day course of linezolid was selected as the comparator in this registrational trial because it is a standard of care for gram-positive HABP/VABP and is consistent with the approved dosage, frequency, and duration [20, 23, 24].

## METHODS

### Study Design and Participants

Protocol MK-1986-002 (Ventilated Pneumonia Treatment with Tedizolid Phosphate and Linezolid [VITAL]) was a randomized, double-blind, double-dummy, phase 3, noninferiority trial conducted at 122 global study sites in 32 countries from June 2014 to June 2018. A scientific advisory committee comprising external and Merck & Co, Inc (Kenilworth, New Jersey), scientists contributed to the development of the study protocol. The study was conducted in accordance with principles of Good Clinical Practice and was approved by the appropriate institutional review boards and regulatory agencies. Written informed consent was provided by a legally acceptable representative before study enrollment.

Eligible patients included intubated and mechanically ventilated adults (aged ≥18 years) diagnosed with ventilated HABP (vHABP) or VABP likely caused by gram-positive cocci. Pneumonia diagnosis was based on the following radiographic and clinical criteria: chest radiograph showing new or progressive infiltrate(s) suggestive of bacterial pneumonia, purulent respiratory secretions, and ≥1 other clinical criterion (fever [≥38°C] or hypothermia [≤35°C], peripheral white blood cells ≥10 000 cells/mm^3^ or leukopenia [≤4500 cells/mm^3^], or ≥15% immature neutrophils). A vHABP diagnosis was made in patients who were intubated and mechanically ventilated after meeting clinical and radiographic criteria for HABP, were hospitalized (including in long-term care facilities) for ≥48 hours or had been discharged from a hospital ≤7 days before, and had ≥1 of the following signs or symptoms present ≤24 hours before intubation: new or worsening cough; dyspnea, tachypnea, or respiratory rate of >30 breaths per minute; and/or hypoxemia. A VABP diagnosis was assigned to patients with clinical signs and symptoms of pneumonia who had ≥48 hours of mechanical ventilation for a noninfectious reason. To meet the case definition of VABP, the protocol also required acute changes in the ventilator support system to enhance oxygenation. Lower respiratory samples had to meet the following microbiologic criteria: Gram stain (performed ≤36 hours before first study drug infusion) of purulent sputum, endotracheal aspirate/respiratory specimen obtained by specimen brush, bronchoalveolar lavage, mini-bronchoalveolar lavage, or exudative pleural effusion demonstrating gram-positive bacteria (with/without gram-negative bacteria); culture from lower respiratory sample obtained ≤72 hours before the first study drug infusion positive for methicillin-resistant *S. aureus* (MRSA); or a MRSA-positive rapid molecular diagnostic test. Key exclusion criteria are listed in the Supplementary Methods.

### Randomization, Stratification, and Blinding

Eligible patients, stratified by geographic region, age (18–64 years and ≥65 years), and underlying diagnosis (trauma or nontrauma), were randomized 1:1 to tedizolid phosphate or linezolid (Supplementary Figure 1) via an interactive voice response system. Patients, the study sponsor, site investigators, and study staff involved in clinical care/evaluations were all blinded to treatment assignments. Staff responsible for study drug inventory, accountability, and preparation, as well as the study monitor at each site, remained unblinded. Masking with tamper-evident material was applied to infusion bags and tubing; after use, infusion bags were returned to a secure location accessible only to unblinded staff.

### Procedures

Randomized patients received tedizolid phosphate 200 mg once daily as a 60-minute IV infusion for 7 days or linezolid 600 mg twice daily as a 60-minute IV infusion for 10 days (patients in either group with concurrent gram-positive bacteremia received 14-day treatment). Patients received matching placebo infusions unique to each active treatment (Supplementary Table 1). No dose adjustments were permitted. Adjunctive gram-negative therapy with investigator-selected standard of care was administered to patients, as needed, based on Gram stain results or a rapid diagnostic test and patient and site epidemiology, with gram-negative adjunctive therapy adjustments made by the blinded investigator.

A central laboratory confirmed the identification and susceptibility testing of bacterial isolates obtained from the infection site or blood using Clinical and Laboratory Standards Institute and European Committee on Antimicrobial Susceptibility Testing susceptibility criteria [25, 26]. Whole blood samples were collected for pharmacokinetic analysis to determine the concentration of tedizolid in plasma using protein precipitation extraction followed by high-performance liquid chromatography–tandem mass spectrometry.

### Outcomes

The primary efficacy end points were day 28 ACM and investigator-assessed clinical response (criteria defined in Supplementary Table 2) at the test-of-cure (TOC) visit (7–14 days after last study drug infusion or time of failure) in the intention-to-treat (ITT) population (all randomized patients). Briefly, clinical cure at TOC was defined for surviving patients as complete resolution of most or all clinical signs and symptoms of vHABP/VABP that were present at baseline, no new signs/symptoms or complications attributable to vHABP/VABP, and no additional antibacterial therapy administered for vHABP/VABP or gram-positive bacteremia except for adjunctive therapy that was given for 14 days. Secondary end points included investigator-assessed clinical response at TOC in the clinically evaluable population (patients who received study drug, had no major confounding events or factors as detailed in the statistical analysis plan, and had evaluable clinical outcomes at the TOC visit; revised from a primary to a secondary end point at protocol amendment); day 28 ACM in the microbiological ITT (mITT) population (patients who received study drug and had confirmed gram-positive pathogen(s) and evaluable clinical outcomes at the TOC visit); investigator-assessed clinical response at TOC in patients with methicillin-susceptible *S. aureus* (MSSA) or MRSA (mITT population); evaluation of safety based on adverse event rates and abnormal laboratory values; and assessment of the pharmacokinetic/pharmacodynamic (PK/PD) profile of tedizolid.

### Pharmacokinetic and Pharmacodynamic Analyses

A previously developed population pharmacokinetic (popPK) model was updated to include PK data from participants in this phase 3 HABP/VABP trial [27]. Individual patient exposures were estimated using the popPK model, and the free fraction of the area under the tedizolid concentration curve over the minimum inhibitory concentration (*f*AUC/MIC) values were calculated to determine the attainment of the PK/PD target of *f*AUC/MIC = 3. This PK/PD target has previously been used to assess the probability of target attainment in patient plasma and was validated in a mouse lung infection model [27, 28].

### Statistical Analyses

This study was designed to determine the noninferiority of tedizolid to linezolid for day 28 ACM in the ITT population using a 10% noninferiority margin and was sufficiently powered to assess clinical cure at TOC in the ITT population using a 12.5% noninferiority margin. A sample size of 726 randomized patients (363 per group) was selected to provide 92% power at a 1-sided significance level of 0.025, assuming a day 28 ACM rate of 20% in both groups. With an evaluability rate of 80%, 726 patients randomized to the ITT population was estimated to provide 87% power to determine noninferiority for clinical cure at TOC, assuming a 50% investigator-assessed clinical cure rate and a 12.5% noninferiority margin. Differences between treatment groups were assessed using 2-sided 95%/97.5% confidence intervals (CIs) calculated using the Miettinen and Nurminen method, with a lower limit greater than –10% considered noninferior for day 28 ACM and a lower limit greater than –12.5% considered noninferior for investigator-assessed clinical cure at TOC [29].

Descriptive statistics were calculated for all categorical and continuous data. For the remaining secondary end points, 2-sided 95% CIs were calculated for the difference in proportions between treatment groups using the Miettinen and Nurminen method. Differences in baseline characteristics between treatment groups were analyzed using the Fisher exact test for dichotomous variables and the Wilcoxon rank sum test for ordinal and continuous variables [30, 31].

### Post Hoc Logistic Regression Model

Predictor factors (variables) were determined using the area under the receiver operating characteristics curve. Backward elimination was performed with entry and stay criteria of 0.3. We used a goodness-of-fit test to quantify the multivariable model calibration, and the factors that best qualified to remain in the model were determined for each treatment group. The test summary of each model parameter was based on the Wald χ ^2^*P* value. All factors with *P* < .05 were considered significant for predicting probability of clinical cure in the tedizolid or linezolid treatment groups.

## RESULTS

### Patients and Pathogens

**Figure 1.**
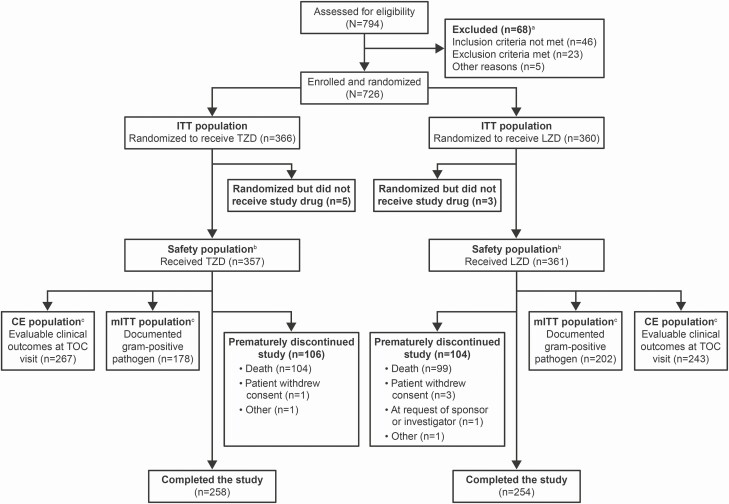
Patient disposition. Abbreviations: CE, clinically evaluable; ITT, intention-to-treat; LZD, linezolid; mITT, microbiological intention-to-treat; TOC, test of cure; TZD, tedizolid. ^a^Patients may have been excluded for multiple reasons. ^b^Four patients were randomized to receive tedizolid phosphate but were administered linezolid in error. These patients were included in the tedizolid ITT population for efficacy analyses but were included in the linezolid safety population. One patient was randomized to receive linezolid but was administered tedizolid phosphate in error. This patient was included in the linezolid ITT population for efficacy analyses but was included in the tedizolid safety population. ^c^Reasons for exclusion from these populations are provided in the Supplementary Appendix (Supplementary Table 7).

A total of 726 patients were enrolled and randomized (tedizolid, n = 366; linezolid, n = 360) at 122 study sites in 32 countries. Patient disposition is summarized in Figure 1. Demographics and baseline characteristics of the ITT population were similar between treatment groups (Table 1). Baseline Acute Physiology and Chronic Health Evaluation (APACHE II) scores ≥20 were reported in 48.4% and 43.1% of patients in the tedizolid and linezolid groups, respectively; median Clinical Pulmonary Infection Scores were 9.0 (range, 3.0–13.0) for both groups at baseline. Within 72 hours before study drug initiation, 322 patients (88.0%) in the tedizolid group and 328 (91.1%) in the linezolid group had received systemic antibacterial therapy. Overall, 295 (80.6%) and 293 (81.4%) patients in the tedizolid and linezolid groups, respectively, received any adjunctive gram-negative therapy (Table 1).

**Table 1.** Patient Demographics and Baseline Characteristics: Intention-to-Treat Population

Characteristic	Tedizolid (n = 366)	Linezolid (n = 360)	*P* Value
Median age (range), years	61.0 (18.0–93.0)	61.0 (18.0–91.0)	.734
Age group, n (%), years			.820
<65	221 (60.4)	214 (59.4)	
≥65	145 (39.6)	146 (40.6)	
Male, n (%)	249 (68.0)	254 (70.6)	.470
Race, n (%)			.304
Asian	68 (18.6)	70 (19.4)	
Black or African American	3 (0.8)	11 (3.1)	
White	269 (73.5)	258 (71.7)	
Other	26 (7.1)	21 (5.8)	
Hispanic or Latino, n (%)	65 (17.8)	61 (16.9)	.934
Median body mass index (range), kg/m^2^	25.5 (13.6–54.7)	26.1 (13.5–49.0)	.398
Geographic region, n (%)			NE
China and Taiwan	12 (3.3)	12 (3.3)	
Europe	193 (52.7)	191 (53.1)	
Latin America	63 (17.2)	64 (17.8)	
Middle East/Africa	26 (7.1)	23 (6.4)	
North America	19 (5.2)	18 (5.0)	
Other Asia Pacific^a^	53 (14.5)	52 (14.4)	
Underlying diagnosis (stratification), n (%)			
Trauma	83 (22.7)	79 (21.9)	.859
Nontrauma	283 (77.3)	281 (78.1)	
Diagnosis, n (%)			
Ventilated hospital-acquired bacterial pneumonia	97 (26.5)	94 (26.1)	.933
Ventilator-associated bacterial pneumonia	269 (73.5)	266 (73.9)	
Diabetes, n (%)	74 (20.2)	86 (23.9)	.245
Gram-positive bacteremia at baseline, n (%)	12 (3.3)	16 (4.4)	.446
Prior antibacterial therapy within 72 hours before first infusion of study drug, n (%)	322 (88.0)	328 (91.1)	ND
β-lactam/β-lactamase inhibitor combination agents	124 (33.9)	103 (28.6)	ND
Third-generation cephalosporins	73 (19.9)	83 (23.1)	ND
Carbapenems	74 (20.2)	79 (21.9)	ND
Glycopeptides (vancomycin and teicoplanin)	69 (18.9)	57 (15.8)	ND
Duration of mechanical ventilation before first dose of study drug, n (%), days			
<5	158 (43.2)	175 (48.6)	.178
≥5	203 (55.5)	182 (50.6)	
Missing	5 (1.4)	3 (0.8)	
Duration of hospitalization before first dose of study drug, n (%), days			
<5	31 (8.5)	25 (6.9)	.487
≥5	330 (90.2)	332 (92.2)	
Missing	5 (1.4)	3 (0.8)	
Partial pressure of oxygen/fraction of inspired oxygen ratio, n (%)			.934
<240	254 (69.4)	246 (68.3)	
≥240	105 (28.7)	104 (28.9)	
Missing	7 (1.9)	10 (2.8)	
Acute Physiology and Chronic Health Evaluation II score			
Median (range)	19.0 (4.0–38.0)	18.0 (4.0–55.0)	.669
<20, n (%)	188 (51.4)	202 (56.1)	.245
≥20, n (%)	177 (48.4)	155 (43.1)	
Glasgow Coma Scale Score, median (range)	8.0 (3.0–15.0)	8.0 (3.0–15.0)	.702
Clinical Pulmonary Infection Score, median (range)	9.0 (3.0–13.0)	9.0 (3.0–13.0)	.050
Sequential Organ Failure Assessment score, median (range)	6.0 (1.0–15.0)	6.0 (1.0–15.0)	.546

Abbreviations: ND, not determined; NE, not estimated.

^a^Includes Australia and New Zealand.

Baseline respiratory pathogens isolated from 380 patients comprising the mITT population (tedizolid, n = 178; linezolid, n = 202) are listed in Supplementary Table 3. *Staphylococcus aureus* was isolated from 166 patients (93.3%) who received tedizolid and 192 (95.0%) who received linezolid; MRSA was isolated from 53 (29.8%) and 66 (32.7%) patients who received tedizolid and linezolid, respectively. Notably, among *S. aureus* isolates (MRSA and MSSA), none had a vancomycin MIC >2 μg/mL and only 10 had an MIC of 2 μg/mL. Overall, 91 (51.1%) and 98 (48.5%) patients who received tedizolid and linezolid, respectively, had mixed gram-negative and gram-positive infections.

In the safety population, 312 patients (87.4%) in the tedizolid group received 5 to 7 doses of tedizolid phosphate and 287 patients (79.5%) in the linezolid group received 15 to 20 doses of linezolid. The mean (standard deviation) treatment duration was 6.4 (1.8) days with tedizolid and 9.1 (2.5) days with linezolid. The mean (standard deviation) duration of mechanical ventilation in the ITT population was 18.4 (10.4) days with tedizolid (n = 366) and 17.1 (10.6) days with linezolid (n = 360).

### Efficacy

The tedizolid day 28 ACM rate was 28.1% vs 26.4% with linezolid (treatment difference, –1.8 (95% CI: –8.2 to 4.7). Tedizolid was noninferior to linezolid based on day 28 ACM in the ITT population, as the lower bound of the 95% CI for the overall treatment difference (linezolid–tedizolid) was above the predefined noninferiority margin of 10% (Table 2). Time to death was similar among the tedizolid and linezolid groups (Supplementary Figure 2). In the mITT population, tedizolid day 28 ACM rates were 23.0% in patients with gram-positive–only infections (monomicrobial and polymicrobial) and 28.6% in patients with gram-positive/gram-negative infections vs 19.2% and 29.6%, respectively, in patients treated with linezolid.

**Table 2.** Primary and Secondary Efficacy Outcomes in Various Patient Populations

Efficacy Outcome	Tedizolid, n/N (%)	Linezolid, n/N (%)	Difference (95% CI; 97.5% CI)
Day 28 all-cause mortality			
Intention-to-treat population	103/366 (28.1)	95/360 (26.4)	–1.8 (–8.2 to 4.7)
Microbiological intention-to-treat population	46/178 (25.8)	49/202 (24.3)	–1.6 (–10.3 to 7.1)
Investigator-assessed clinical response at test of cure			
Intention-to-treat population	206/366 (56.3)	230/360 (63.9)	–7.6 (–14.7 to –.5; –15.7 to 0.5)
Clinically evaluable population	143/267 (53.6)	146/243 (60.1)	–6.5 (–15.1 to 2.1; –16.3 to 3.3)

Abbreviation: CI, confidence interval.

Noninferiority was not demonstrated for tedizolid compared with linezolid based on the investigator-assessed clinical cure rate at TOC in the ITT population; the investigator-assessed clinical cure rate was 56.3% with tedizolid vs 63.9% with linezolid, for a treatment difference of –7.6 (97.5% CI: –15.7 to 0.5). Time to investigator-assessed clinical failure in the ITT population is summarized in Supplementary Figure 3. In the ITT population, the 25% quartile time to investigator-assessed clinical failure was 2.0 (95% CI: 2.0 to 3.0) days in the tedizolid group and 3.0 (95% CI: 2.0 to 11.0) days in the linezolid group. Investigator-assessed clinical response rates at the TOC visit were lower for tedizolid compared with linezolid across most patient subgroups (Supplementary Figure 4).

The day 28 ACM rate in the mITT population was 25.8% with tedizolid vs 24.3% with linezolid. Investigator-assessed clinical cure rates at TOC by baseline pathogens in the mITT population were lower with tedizolid than with linezolid across most pathogens, including MSSA (Table 3).

**Table 3.** Clinical Cure at Test of Cure by Pathogen: Microbiological Intention-to-Treat Population

Clinical Cure by Pathogen^a^	Tedizolid, n/N (%)	Linezolid, n/N (%)	Difference (95% Confidence Interval)
Gram-positive pathogens	96/178 (53.9)	137/202 (67.8)	
*Staphylococcus aureus*	86/166 (51.8)	130/192 (67.7)	–15.9 (–26.0 to –5.8)
Methicillin-resistant *S. aureus*	29/54 (53.7)	45/69 (65.2)	–11.5 (–28.9 to 5.9)
Methicillin-susceptible *S. aureus*	58/117 (49.6)	86/128 (67.2)	–17.6 (–29.8 to –5.4)
*Streptococcus pneumoniae*	13/16 (81.3)	7/10 (70.0)	
Monomicrobial gram-positive pathogens	55/86 (64.0)	77/104 (74.0)	
Mixed infection	42/94 (44.7)	60/98 (61.2)	
*Acinetobacter baumannii* complex	14/30 (46.7)	25/40 (62.5)	
*Escherichia coli*	6/15 (40.0)	6/9 (66.7)	
*Klebsiella pneumoniae*	11/24 (45.8)	14/30 (46.7)	
*Pseudomonas aeruginosa*	8/14 (57.1)	10/14 (71.4)	
Other	21/45 (46.7)	29/40 (72.5)	

^a^Limited to pathogens with ≥10 isolates in 1 treatment group.

### Post Hoc Logistic Regression Model

A post hoc logistic regression model was used to assess potential factors that predict investigator-assessed clinical response in patients with vHABP/VABP treated with tedizolid or linezolid. Predicting factors (see Supplementary Table 4) were evaluated for inclusion in the model. Several factors that remained in the model were significant predictors of clinical response for tedizolid, including APACHE II score, geographic region (North America vs other regions), and baseline pathogen (gram-positive plus gram-negative vs gram-positive only), whereas none of the factors included in the model were significant predictors of clinical response for linezolid (Supplementary Table 5). None of the factors included in the model accounted for the observed difference in investigator-assessed clinical response between treatment groups.

### Pharmacokinetic Analysis

Pharmacokinetic targets were met, and no exposure–response relationship was demonstrated for tedizolid (data not shown). No significant differences in ACM or clinical response were observed between tedizolid exposure quartiles. In addition, nearly all participants who had both clinical response and MIC data available achieved the PK/PD target of *f*AUC/MIC = 3 at the TOC visit (98/101 [97.0%]) and at day 28 (160/164 [97.6%]).

### Safety

Within the safety population, the proportions of patients with any treatment-emergent adverse events (TEAEs), serious TEAEs, and TEAEs that led to discontinuation of study drug were comparable between groups (Table 4). Investigator-assessed drug-related TEAEs were reported in approximately 8% and 12% of patients who received tedizolid and linezolid, respectively, while drug-related serious TEAEs were infrequent in both groups. The total numbers of deaths reported in the tedizolid and linezolid groups were comparable. None of the deaths were considered related to tedizolid; 1 death in the linezolid group (acute kidney injury) was considered to be study drug related by the investigator. Proportions of patients with hemoglobin levels and neutrophil counts below the lower limit of normal or substantially abnormal were also comparable between groups (Table 5). The proportion of patients with platelet counts below the lower limit of normal was lower with tedizolid than with linezolid (Table 5).

**Table 4.** Adverse Event Rates of the Safety Population

AE Category, n (%)	Tedizolid (n = 357)	Linezolid (n = 361)
Any AE	327 (91.6)	325 (90.0)
Any TEAE	326 (91.3)	325 (90.0)
Drug-related TEAE	29 (8.1)	43 (11.9)
TEAE leading to discontinuation of study drug	4 (1.1)	3 (0.8)
Any TEAE leading to death	101 (28.3)	103 (28.5)
Drug-related TEAE leading to death	0	1 (0.3)
Serious TEAE	129 (36.1)	149 (41.3)
Drug-related serious AE	0	4 (1.1)
Most common drug-related TEAEs^a^		
Anemia	2 (0.6)	4 (1.1)
Thrombocytopenia	2 (0.6)	3 (0.8)
Diarrhea	6 (1.7)	20 (5.5)
Nausea	2 (0.6)	2 (0.6)
Vomiting	2 (0.6)	0
Alanine aminotransferase increased	3 (0.8)	2 (0.6)
Aspartate aminotransferase increased	2 (0.6)	2 (0.6)
Hepatic enzyme increased	2 (0.6)	2 (0.6)
Rash	3 (0.8)	2 (0.6)

Four patients were randomized to tedizolid but received linezolid in error. These 4 patients were included in the linezolid safety population (not the tedizolid safety population). One patient was randomized to linezolid but received tedizolid in error. This patient was included in the tedizolid safety population (not the linezolid safety population).

Abbreviations: AE, adverse event; TEAE, treatment-emergent adverse event.

^a^Limited to drug-related TEAEs recorded in ≥0.5% of patients in the tedizolid treatment group.

**Table 5.** Postbaseline Abnormal Clinical Laboratory Values of the Safety Population

Parameter	Tedizolid (n = 357)	Linezolid (n = 361)
Hemoglobin, n	345	346
Below LLN, n (%)	298 (86.4)	304 (87.9)
Substantially abnormal (<10.1 g/dL [male]; <9 g/dL [female]), n (%)	50 (14.5)	49 (14.2)
Platelet count, n	344	342
Below LLN, n (%)	96 (27.9)	130 (38.0)
Substantially abnormal (<112 × 10^3^/mm^3^), n (%)^a^	38 (11.0)	53 (15.5)
Neutrophil count, n	340	344
Below LLN, n (%)	18 (5.3)	16 (4.7)
Substantially abnormal (<0.8 × 10^3^/mm^3^), n (%)^b^	3 (0.9)	0

Four patients were randomized to tedizolid but received linezolid in error. These 4 patients were included in the linezolid safety population (not the tedizolid safety population). One patient was randomized to linezolid but received tedizolid in error. This patient was included in the tedizolid safety population (not the linezolid safety population).

Abbreviation: LLN, lower limit of normal.

^a^Substantially abnormal was defined as >2 times the upper limit of normal (ULN) for values normal at baseline and >2 times the ULN and >2 times the baseline value for values abnormal at baseline.

## DISCUSSION

In this randomized, controlled trial, we demonstrated that tedizolid was noninferior to linezolid for day 28 ACM in the treatment of patients with gram-positive vHABP/VABP. Use of day 28 ACM as the primary end point was consistent with guidance from the FDA [24]. ACM rates were consistently similar between treatment groups over time and were comparable to previously reported mortality rates in gram-positive HABP/VABP clinical trials [32–34]. However, the result for the key secondary end point in this study does not support the primary end point, as investigator-assessed clinical outcome did not meet the non-inferiority criterion. HABP/VABP is a complex disease process, and it is possible that neither day 28 ACM nor investigator-assessed clinical response at TOC alone adequately captures the clinical benefit of antibacterial therapies in this patient population. Noninferiority assessment in HABP/VABP trials is further complicated by the use of investigator-assessed clinical response as a primary/key secondary end point, as it remains a subjective end point with no consensus definition for clinical cure [35]. No single factor examined in subgroup analyses or in the post hoc logistic regression model accounted for the imbalance in investigator-assessed clinical cure rates between groups. Clinical cure rates in the linezolid group were consistent with previous data [34]. The high proportions of patients with TEAEs in both groups was anticipated because patients were critically ill, and were consistent with previous studies for other antibacterial agents conducted in patients with HABP/VABP [34, 36–38]. Few TEAEs led to study drug discontinuation, none of the tedizolid-related AEs were serious, and none of the tedizolid-related TEAEs led to death. TEAEs in the tedizolid group were generally consistent with previous studies in patients with ABSSSIs [17, 18, 39]. An exposure–response relationship was not expected. The difference in clinical outcome is unlikely to be due to insufficient dosing, as previous studies have demonstrated that tedizolid has excellent pulmonary penetration with ELF concentrations higher than free plasma concentrations for the entire dosing interval and an approximately 40:1 ELF-to-plasma penetration ratio [19]. In addition, the high rates of PK/PD target attainment at TOC and day 28 (97%) also suggest that insufficient dosing was not an issue in this population.

The imbalance in clinical cure rates between groups may result from the interplay of several confounding factors. To assess this possibility, we examined factors in each group that may potentially predict clinical response (eg, partial pressure of oxygen to fraction of inspired oxygen ratio, APACHE II score, renal function, diabetes, duration of ventilation before first dose) to determine whether differences in these factors would corroborate the clinical assessment. No single factor nor combination of factors adequately accounted for the observed imbalance in clinical cure rates between groups.

The difference in mean treatment duration between treatment groups (6.4 days for tedizolid vs 9.1 days for linezolid) was a result of the study design, with the shorter course of tedizolid prescribed by current guidelines and the longer course of linezolid consistent with the approved dosing regimen that was necessary for use in a clinical trial [20, 24]. Although it remains possible that the difference in treatment duration contributed to the outcome differences between treatment groups, previous studies showed no difference in mortality or relapse with short-course vs long-course antibacterial therapy for HABP/VABP [21, 22]. This study was limited by the number of patients with vHABP/VABP caused by documented gram-positive pathogens; however, the size of this population is comparable to those included in other gram-positive HABP/VABP clinical trials [32, 34]. A large proportion of gram-positive pathogens were identified in mixed microbial infections (51.1% and 48.5% of patients in the tedizolid and linezolid groups, respectively), and gram-negative pathogen susceptibilities were unavailable to confirm adequacy of adjunctive therapy. Together, these limitations make it difficult to determine whether efficacy (or lack of efficacy) was primarily attributable to the gram-positive therapy, gram-negative therapy, or both.

In summary, tedizolid was noninferior to linezolid for day 28 ACM, but noninferiority was not demonstrated for investigator-assessed clinical response. Tedizolid was generally well tolerated for up to 14 days in adult patients with vHABP/VABP. No new safety concerns were observed.

## Supplementary Data

Supplementary materials are available at *Clinical Infectious Diseases* online. Consisting of data provided by the authors to benefit the reader, the posted materials are not copyedited and are the sole responsibility of the authors, so questions or comments should be addressed to the corresponding author.

ciab032_suppl_Supplementary_Material

## Notes

***Author contributions.*** All authors are responsible for the work described here. All authors were involved in at least 1 of the following: conception, design of work, or acquisition, analysis, interpretation of data and drafting the manuscript and/or revising/reviewing the manuscript for important intellectual content. All authors provided final approval of the version to be published. All authors agree to be accountable for all aspects of the work in ensuring that questions related to the accuracy or integrity of any part of the work are appropriately investigated and resolved.

***Acknowledgments.*** We thank the patients and their families and caregivers for participating in this study, along with all investigators and site personnel. A full list of primary investigators is provided in Supplementary Table 6. Medical writing and/or editorial assistance was provided by Todd Waldron, PhD, CMPP, of The Lockwood Group, Stamford, Connecticut. This assistance was funded by Merck Sharp & Dohme Corp, a subsidiary of Merck & Co, Inc, Kenilworth, NJ, USA (MSD).

***Financial support.*** This work was supported by MSD.

***Potential conflicts of interest.*** J. R. B., N. B., J. Y. K., and C. D. A. are employees of MSD who may own stock and/or hold stock options in Merck & Co, Inc. M. W. P. was previously an employee of MSD who may own stock and/or hold stock options in Merck & Co, Inc. R. G. W. reports grants and personal fees from MSD during the conduct of the study; grants and personal fees from Arsanis, Melinta, Polyphor, Shionogi, and the Medicines Company; personal fees from KBP Biosciences, Meiji Seika, and Microbiotix outside the presented work; and has received institutional research funding from MSD. A. R. reports grants and personal fees from MSD and from bioMerieux during the conduct of the study. D. R. G. reports grants and personal fees from MSD during the conduct of the study and from Bayer and Janssen outside the presented work. The remaining authors: No reported conflicts of interest. All authors have submitted the ICMJE Form for Disclosure of Potential Conflicts of Interest. Conflicts that the editors consider relevant to the content of the manuscript have been disclosed.
