# Supplementary material for: A Phase 3, Randomized, Double-Blind Study Comparing Tedizolid Phosphate and Linezolid for Treatment of Ventilated Gram-Positive Hospital-Acquired or Ventilator-Associated Bacterial Pneumonia
Source: Clin Infect Dis. 2021 Mar 15;73(3):e710–8. doi: 10.1093/cid/ciab032 (PMC8326538; doi:10.1093/cid/ciab032)
Supplement: ciab032_suppl_Supplementary_Material [file ciab032_suppl_supplementary_material.docx]

# SUPPLEMENTARY APPENDIX.

**Supplementary Methods**

Key exclusion criteria included known or suspected community-acquired bacterial pneumonia or viral, fungal, or parasitic pneumonia; *Legionella pneumophila* pneumonia; cystic fibrosis; bronchiectasis; HIV infection with CD4 count <200 cells/mm^3^; known or suspected *Pneumocystis jirovecii* pneumonia or active tuberculosis; lung abscess; evidence of endocarditis; tracheobronchitis (if no evidence of pneumonia); receipt of systemic or inhaled antibacterial therapy effective for gram-positive vHABP/VABP pathogens for >24 hours in the prior 72 hours (exceptions included disease progression after >48 hours or development of new vHABP/VABP symptoms and a new infiltrate on the prior therapy, receipt of systemic antibacterial therapy that did not cover the gram-positive pathogen isolated from respiratory culture, and antibacterial therapy for gut decontamination or gut motility); administration of tedizolid phosphate or linezolid ≤30 days before the first study drug infusion (except for a single administration of linezolid within 24 hours of first study drug infusion); recent opportunistic infection with underlying cause still present (eg, leukemia, transplant, acquired immunodeficiency syndrome); and severe renal disease requiring peritoneal dialysis (hemodialysis, venovenous dialysis, and other forms of renal filtration were not excluded).

**Supplementary Table S1.** **Daily Dose Schedule by Treatment Group and Bacteremia Status**

|  |  | No Bacteremia | | Gram-positive Bacteremia | |
| --- | --- | --- | --- | --- | --- |
|  | Dose^a^ | TZD Group | LZD Group | TZD Group | LZD Group |
| Days 1–10 | Odd  (1–19) | Infusion A: Active TZD  Infusion B: Placebo LZD | Infusion A: Placebo TZD  Infusion B: Active LZD | Infusion A: Active TZD  Infusion B: Placebo LZD | Infusion A: Placebo TZD  Infusion B: Active LZD |
|  | Even  (2–20) | Placebo LZD | Active LZD | Placebo LZD | Active LZD |
| Days 11–14 | Odd  (21–27) |  |  | Infusion A: Active TZD  Infusion B: Placebo LZD | Infusion A: Placebo TZD  Infusion B: Active LZD |
|  | Even (22–28) |  |  | Placebo LZD | Active LZD |

Abbreviations: IV, intravenous; LZD, linezolid infusion bag (300 mL); TZD, tedizolid infusion bag (250 mL).

^a^For odd doses, patients received an initial IV infusion of 250-mL tedizolid or tedizolid placebo, followed by IV line flushing, followed by a second IV infusion of 300-mL linezolid or linezolid placebo; for even doses (12 ± 2 hours after an odd dose), patients received a 300-mL IV infusion of linezolid or linezolid placebo.

**Supplementary** Table S2. **Definitions Used in Investigator’s Assessment of Clinical Response**

| Visit | Clinical Outcome | Definition |
| --- | --- | --- |
| End of treatment and test of cure |  |  |
|  | Cure | - Complete resolution of most or all clinical signs and symptoms of vHABP/VABP that were present at baseline, AND |
|  |  | - No new signs/symptoms or complications attributable to vHABP/VABP, AND |
|  |  | - No additional antibacterial therapy administered for vHABP/VABP or gram-positive bacteremia except for adjunctive therapy that was given for 14 days, AND |
|  |  | - Patient is alive |
|  | Failure | - Progression, relapse, or recurrence of new symptoms or complications attributable to vHABP/VABP or gram-positive bacteremia due to the same pathogen isolated at baseline, OR |
|  |  | - Lack of resolution (persistence) or insufficient improvement in signs/symptoms of vHABP/VABP that were present at baseline that required new or prolonged antibacterial therapy, OR |
|  |  | - Patient died from any cause |
|  | Indeterminate | Study data were not available for the evaluation of efficacy for any reason including: |
|  |  | - Diagnosis of gram-negative vHABP/VABP with no gram-positive pathogen isolated within 5 days after randomization |
|  |  | - Lost to follow-up |
|  |  | - Withdrawal of consent |
|  |  | - Insufficient clinical documentation that precluded the classification of clinical outcome of vHABP/VABP |
|  |  | - Extenuating circumstances that precluded the classification of clinical outcome of vHABP/VABP |
| Late follow-up |  |  |
|  | Sustained clinical cure | - Complete resolution, marked improvement, or return to baseline of all signs, AND |
|  |  | - Symptoms of pneumonia and improvement or lack of progression of all chest x-ray abnormalities, such that no additional antibacterial therapy was required for treatment of the current infection |
|  | Relapse | - Recurrence of signs or symptoms of pneumonia or new radiographic evidence of pneumonia or death due to pneumonia or pneumonia-related complications in a patient assessed as cured at the test-of-cure visit |
|  | Indeterminate | Study data were not available for the evaluation of efficacy for any reason including: |
|  |  | - Lost to follow-up |
|  |  | - Withdrawal of consent |
|  |  | - Death unrelated to pneumonia |
|  |  | - Extenuating circumstances that precluded the classification as cure or relapse |

Abbreviations: VABP, ventilator-associated bacterial pneumonia; vHABP, ventilated hospital-acquired bacterial pneumonia.

**Supplementary** Table S3. **Baseline Respiratory Tract Pathogens Isolated in the Microbiological Intention-to-Treat Population**

| Pathogen, n (%) | Tedizolid  (n=178) | Linezolid  (n=202) |
| --- | --- | --- |
| Gram-positive organisms | 178 (100.0) | 202 (100.0) |
| *Staphylococcus aureus* | 166 (93.3) | 192 (95.0) |
| MRSA | 53 (29.8) | 66 (32.7) |
| MSSA | 116 (65.2) | 126 (62.4) |
| *Streptococcus pneumoniae* | 16 (9.0) | 10 (5.0) |
| *Streptococcus pyogenes* (Group A) | 0 | 1 (0.5) |
| Concomitant gram-negative organisms^a^ | 91 (51.1) | 98 (48.5) |
| *Acinetobacter baumannii* complex | 29 (16.3) | 38 (18.8) |
| *Klebsiella pneumoniae* | 21 (11.8) | 29 (14.4) |
| *Pseudomonas aeruginosa* | 13 (7.3) | 14 (6.9) |
| *Escherichia coli* | 14 (7.9) | 9 (4.5) |
| *Haemophilus influenzae* | 9 (5.1) | 9 (4.5) |
| *Proteus mirabilis* | 7 (3.9) | 3 (1.5) |
| Monomicrobial infections | 86 (48.3) | 104 (51.5) |
| *Staphylococcus aureus* | 83 (46.6) | 100 (49.5) |
| MRSA | 24 (13.5) | 40 (19.8) |
| MSSA | 59 (33.1) | 60 (29.7) |
| *Streptococcus pneumoniae* | 3 (1.7) | 3 (1.5) |
| *Streptococcus pyogenes* (Group A) | 0 | 1 (0.5) |

Abbreviations: MRSA, methicillin-resistant *Staphylococcus aureus*; MSSA, methicillin-susceptible *Staphylococcus aureus*.

^a^Limited to pathogens with ≥10 total isolates across treatment groups.

**Supplementary** Table S4. Covariates Included in the Logistic Regression Model

| Covariate | Correlated Factors |
| --- | --- |
| Age (<65 vs ≥65 y) | APACHE II score, renal function, and SOFA score |
| Country | Region |
| vHABP vs VABP | PaO_2_/FiO_2_ ratio, APACHE II score, SOFA score, and CPIS |
| APACHE II score (<20 vs ≥20) | Age, renal function (baseline CrCL), PaO_2_/FiO_2_ ratio, CPIS, and SOFA score |
| Renal function (baseline CrCL) | Age, BMI, and SOFA score |
| Gram-negative adjunctive therapy coverage (presumed adequate: yes vs no)^a^ | Monomicrobial/mixed microbial, baseline pathogen MRSA, and MSSA |
| PaO_2_/FiO_2_ ratio at baseline (≤250 mmHg vs >250 mmHg) | CPIS, SOFA score, and APACHE II score |
| Diabetes (yes vs no) | BMI |
| BMI, kg/m^2^  <18.5  18.5–24.9  25.0–29.9  30.0–34.9  ≥35 | Age |
| Duration of hospitalization before first dose | vHABP vs VABP |
| Duration of ventilation before first dose | vHABP vs VABP |
| Baseline pathogen (MRSA vs MSSA) | Monomicrobial/mixed microbial |
| Gram-positive only vs gram-negative only vs gram-positive plus gram-negative | Baseline pathogen (MRSA vs MSSA) |
| Selected gram-negative species:  *Acinetobacter baumannii* complex  *Pseudomonas aeruginosa*  *Klebsiella pneumonia* | Monomicrobial/mixed microbial |

Abbreviations: APACHE, Acute Physiology and Chronic Health Evaluation; BMI, body mass index; CPIS, Clinical Pulmonary Infection Score; CrCL, creatinine clearance; FiO_2_, fraction of inspired oxygen; MRSA, methicillin-resistant *Staphylococcus aureus*; MSSA, methicillin-susceptible *Staphylococcus aureus*; PaO_2_, partial pressure of oxygen; SOFA, Sequential Organ Failure Assessment; VABP, ventilator-associated bacterial pneumonia; vHABP, ventilated hospital-acquired bacterial pneumonia.

^a^Gram-negative pathogen susceptibilities were unavailable to confirm adequacy of adjunctive therapy; adjunctive gram-negative therapy was considered appropriate if the selected agents were considered a preferred agent for the isolated pathogen according to treatment guidelines.

**Supplementary Table S5. Factors Predicting Clinical Success With Tedizolid or Linezolid Treatment for vHABP/VABP**

| Odds Ratio Estimate^a^ | Lower Confidence Limit | Upper Confidence Limit | Predicting Factor | *P* Value^b^ |
| --- | --- | --- | --- | --- |
| **Tedizolid** | | | | |
| 0.266 | 0.128 | 0.553 | APACHE II score (<20 vs ≥20) | .0004 |
| 2.180 | 0.861 | 5.519 | Diabetes (yes vs no) | .1001 |
| 1.098 | 0.254 | 4.752 | Geographic region (Europe vs North America) | .2423 |
| 1.019 | 0.209 | 4.961 | Geographic region (Latin America vs North America) | .2495 |
| 5.099 | 1.009 | 25.775 | Geographic region (North America vs other regions) | .0027 |
| 0.466 | 0.233 | 0.931 | Baseline pathogen (gram-positive plus gram-negative vs gram-positive only) | .0307 |
| **Linezolid** | | | | |
| 0.553 | 0.274 | 1.118 | Diabetes (yes vs no) | .0991 |
| 0.566 | 0.295 | 1.087 | Baseline pathogen (gram-positive plus gram-negative vs gram-positive only) | .0875 |
| 0.468 | 0.230 | 0.953 | Renal impairment (mild/moderate vs normal) | .1365 |
| 0.780 | 0.221 | 2.758 | Renal impairment (severe/on dialysis vs normal) | .8349 |

Abbreviations: APACHE, Acute Physiology and Chronic Health Evaluation; VABP, ventilator-associated bacterial pneumonia; vHABP, ventilated hospital-acquired bacterial pneumonia.

^a^An odds ratio of 1 indicates that clinical success is equally likely to occur in patients from both predictor factors categories, an odds ratio >1 indicates that the clinical success is more likely to occur in patients from the predictor factor category listed second, and an odds ratio <1 indicates that the clinical success is less likely to occur in patients from the predictor factor category listed second.

^b^Obtained using Wald chi-square.

**Supplementary Table S6. Study Investigators**

| Primary Investigator | Patients Randomized, n |
| --- | --- |
| Australia |  |
| Martin Sterba Level 2, Block A, Loftus Street Wollongong, NSW, 2500 | 2 |
| Belarus |  |
| Ihar Adzerikho  1 Lesnoy Agrogorodok  Minsk, Minsk district, 223041 | 5 |
| Elena Mikhailova  5 Bratiev Lizyukovyh St.  Gomel, 246029 | 4 |
| Aliaksandr Skrahin  157 Dolginovski Trakt  Gomel, 246027 | 2 |
| Belgium |  |
| Pieter Depuydt  De Pintelaan 185  Gent, 9000 | 4 |
| Brazil |  |
| Suzana Margareth Ajeje Lobo  Av Brigadeiro Faria Lima 5544  2 Andar Sao Jose do Rio Preto  Sao Paulo, 15090-000 | 16 |
| Maria Patelli Juliani Souza Lima  Av John Boyd Dunlop s/n Campinas  SP, 13060-904 | 8 |
| Antonio Tarcisio de Faria Freire  Av. Francisco Sales 1111  1 Andar Unidade de Pesquisa Clinica Belo Horizonte  MG, 30150-321 | 28 |
| Alvaro Rea-Neto, M.D.  Av. Republica Argentina, 4406 UTI geral Curitiba  PR, 81050-000 | 1 |
| Canada |  |
| Paul Boucher  3134 Hospital Drive  N.W. Calgary, AB, T2N 2T9 | 2 |
| Germain Poirier  3120 Boul. Taschereau Room E-311  Greenfield Park, QC, J4V 2H1 | 1 |
| China |  |
| Yongchang Sun  No. 1 East Jiaoming Xiang  Dongcheng Beijing, Beijing, 100730 | 2 |
| Xiangyan Zhang  No. 83 East Zhongshan Road  Guiyang, Guizhou, 550002 | 1 |
| Jianying Zhou  No. 79 Qingchun Road EC office  Hangzhou, Zhejiang, 310003 | 1 |
| Yijiang Huang  No. 19 Xiuhua Road  Xiuying District Haikou, 570311 | 1 |
| Kejing Ying  No. 3 Qinchun East Road  Hangzhou, Zhe Jiang, 310016 | 3 |
| Yimin Li  No. 151 Yanjiang Road  Guangzhou, Guangdong, 510120 | 3 |
| Ping Chen  No. 139 The People Road  Changsha, Hunan, 410011 | 3 |
| Mao Huang  No. 300 Guangzhou Road  Nanjing, Jiang Su Province, 210029 | 2 |
| Benquan Wu  No. 600 Tianhe Road  Tianhe District, Guangzhou, 510630 | 1 |
| Guochao Shi  No. 197 Rui Jin Er Road  Shanghai, 200025 | 1 |
| Czech Republic |  |
| Jan Manak Sokolska 581  Klinika onkologie a radioterapie  Hradec Kralove, 500 05 | 4 |
| Jan Pachl  Srobarova 50  Praha, 100 34 | 9 |
| Martin Smrcka Jihlavska  20 Interni hematologicka onkologicka klinika  Brno, 625 00 | 2 |
| Estonia |  |
| Ulo Kivistik Sutiste  19 Tallinn, 13419 | 7 |
| France |  |
| Marc Clavel/Bruno Francois  2 Av Martin Luther King  Limoges, 87042 | 1 |
| Philippe Seguin  2, rue Henri Le Guilloux  Rennes, 35033 | 5 |
| Gaetan Plantefeve, M.D.  69 Rue Lieutenant Colonel Prudhon  Argenteuil, 95100 | 5 |
| Antoine Roquilly  1 Place Alexis-Ricordeau  Anesthesie Reanimation Chirurgicale  Nantes, 44093 | 11 |
| Sigismond Lasocki  4 rue Larrey  Pole Anesthesie - Reanimation  Angers, 49933 | 5 |
| Georgia |  |
| Revaz Tabukashvili  9 Tsinandali St.  Tbilisi, 0144 | 6 |
| George Ingorokva  9 Tsinandali St.  Tbilisi, 0144 | 17 |
| Nikoloz Kartsivadze  18/20 Lubliana St.  Tbilisi, 0159 | 39 |
| Irakli Panchulidze/Mamuka Nemsitsveridze  83A Javakhishvili St.  Kutaisi, 4600 | 8 |
| Manana Makhviladze  16 Al. Kazbegi Prosp.  Tbilisi, 0160 | 2 |
| Sophio Maglaperidze/Jano Vashadze/Zviad Bakhtadze  2 Otskheli str.  Kutaisi, 4600 | 25 |
| Irakli Panchulidze  10 Solomon Pirveli St.  Kutaisi, 4600 | 16 |
| Vakhtang Kaloiani  1 Chachava St.  Tbilisi, 0159 | 11 |
| Germany |  |
| Sven Bercker  Liebigstrasse 20  Leipzig, 04103 | 4 |
| Ulrich Jaschinski  Stenglinstrasse 2  Augsburg, 86156 | 1 |
| Greece |  |
| Spyros Zakynthinos  45-47 Ipsilandou Str.  Athens, 10676 | 1 |
| Guatemala |  |
| Luis Demetrio Gonzalez Patzan  Finca El Palomar Acatan  Santa Rosita, 01016, Zona 16 | 27 |
| Israel |  |
| Galia Rahav  Tel Hashomer  Ramat Gan, 5265601 | 8 |
| Ran Nir-Paz  PO Box 12000  Jerusalem, 9112001 | 1 |
| Yaron Bar Lavie, M.D.  Ha'aliya Hashni’ya St 8  PO Box 9602  Haifa, 31096 | 17 |
| Japan |  |
| Nobuhiro Inagaki  1509-2, Oaza-miyazaki  Oita, 870-1195 | 1 |
| Satoshi Fujimi  3-1-56, Bandaihigashi, Sumiyoshi-ku  Osaka, 558-8558 | 12 |
| Takeshi Takahashi  1-5, Ninomaru, Chuo-ku  Kumamoto, 860-0008 | 3 |
| Kazuhiko Yamada  1-21-1, Toyama, Shinjuku-ku  Tokyo, 162-8655 | 2 |
| Shigeyuki Tamura/Yutaka Takeda  3-1-69, Inabasou  Amagasaki, Hyogo, 660-8511 | 4 |
| Eiji Kawamoto  2-174, Edobashi  Tsu, Mie, 514-8507 | 2 |
| Ryosuke Tsuruta  1-1-1, Minamikogushi  Ube, Yamaguchi, 755-8505 | 2 |
| Takaaki Kikuno  2-5-1, Higashigaoka, Meguro-ku  Tokyo, 152-8902 | 7 |
| Michinori Shirano/Yu Kasamatsu  2-13-22, Miyakojimahondori, Miyakojima-ku  Osaka-shi, Osaka, 534-0021 | 1 |
| Mihoko Nakajima/Tomotsugu Nakano  2-34-10 Ebisu, Shibuya-ku  Tokyo, 150-0013 | 1 |
| Shinsuke Fujiwara  2436, Oaza-shimojukuhei, Ureshinocho  Ureshino, Saga, 843-0393 | 1 |
| Taisuke Kitamura/Tsuyoshi Nojima  2125-1, Ike  Kochi, 781-8555 | 2 |
| Yuji Nakamura  2-7-9, Nishihonmachi  Shobara, Hiroshima, 727-0013 | 1 |
| Yusuke Koizumi  1-1, Yazakokarimata  Nagakute, Aichi, 480-1195 | 4 |
| Tsukasa Kuwana  30-1, Oyaguchi, Kami-cho,  Itabashi-ku, Tokyo, 173-8610 | 5 |
| Yuichi Fukuda  9-3, Hirasecho  Sasebo, Nagasaki, 857-8511 | 1 |
| Kazuki Konishi  64-9, Tsunagi-azaoirino  Morioka-shi, Iwate, 020-0055 | 1 |
| Takefumi Saito  825, Terunuma, Tokai-mura  Naka-gun, Ibaraki, 319-1113 | 2 |
| Takahiro Ashikawa  27-1, Takinai-cho  Tanabe, Wakayama, 646-8558 | 1 |
| Kazakhstan |  |
| Murat Zhanuzakov  6 Dzhandosova St.  Almaty, 050040 | 6 |
| Latvia |  |
| Andrejs Levins  Lielvardes str. 68  Riga, 1006 | 5 |
| Lebanon |  |
| Oussaima El Dbouni Bir Hassan  Jnah, Beirut, 1 | 1 |
| Mexico |  |
| Eduardo Rodriguez Noriega  Hospital N° 308, Col. El Retiro  Instituto Patologia Infecciosa  Guadalajara, 44280 | 4 |
| Daniel Rodriguez Gonzalez  Salvador Quevedo y Zubieta 750 Col. Independencia  Unidad de Terapia Intensiva  Guadalajara, 44340 | 24 |
| Adrian Camacho Ortiz  Av. Madero y Gozalitos s/n  Col Mitras Centro  Monterrey, 64460 | 14 |
| Peru |  |
| Maria Edelmira Cruz S.  Av Anselmo Alvarez s/n  Wanchaq Centro de Investigación de Enfermedades Infecciosas y Tropicales  Cuzco, 08006 | 1 |
| Manuel Jesus Mayorga E.  Av Aramburu s/n - cuadra 2  Miraflores Centro de Investigacion  Lima, 15046 | 3 |
| Philippines |  |
| Marie Grace Dawn Tindog Isidro  E. Lopez Street Jaro 5 F Research Room  Iloilo City, 5000 | 7 |
| Lenora Canizares Fernandez  Taft Avenue  Medical Research Laboratories  Manila, 1000 | 29 |
| Joel Santiaguel  Katipunan Road, Corner P. Tuazon Avenue  Quezon City, 1109 | 1 |
| Romania |  |
| Ivars Krastins  Slimnicas Street 25  Bucharest, 050098 | 2 |
| Russia |  |
| Vladimir Simanenkov  2A Kostyushko St.  Saint Petersburg, 196247 | 1 |
| Lyubov Shpagina  21 Polzunova St.  Novosibirsk, 630051 | 25 |
| Yulia Popova  1/1 Velozavodskaya St.  Moscow, 115280 | 2 |
| Alina Agafina  9 Borisova St.  Litera B  Sestroretsk, 197706 | 2 |
| Olga Shevtsova  6 Zalesskogo St.  Novosibirsk, 630047 | 7 |
| Lyudmila Gennadyevna Lenskaya  96 Ivana Chernykh St.  Tomsk Regional Clinical Hospital. 5th floor. 5th block.  Tomsk, 634063 | 35 |
| Tatiana Martynenko  75 Zmeinogorskiy trakt  City Hospital 5. Barnaul. Pulmonology Department  Barnaul, 656045 | 10 |
| Anton Povzun  3 Budapeshtskaya St.  Saint Petersburg, 192242 | 7 |
| Konstantin Apartsin  100 Yubileynyy Mikrorayon  Irkutsk, 664079 | 5 |
| Galina K. Reshedko  40 Frunze St  Smolensk, 214006 | 10 |
| Olga Ershova  11 Zagorodnyy sad St.  Yaroslavl, 150003 | 3 |
| Boris V. Berezhanskiy  1 Fortunatovskaya St.  4 building. 7th floor. Reanimation  Moscow, 105187 | 4 |
| Serbia |  |
| Jovan Matijasevic  Put Doktora Goldmana 4 Clinic for Urgent Pulmonology Sremska Kamenica, 21204 | 4 |
| Ivan Palibrk  Dr Koste Todorovica 6 First Surgical Clinic  Belgrade, 11000 | 7 |
| Vesna Bumbasirevic  Pasterova 2  Belgrade, 11000 | 8 |
| South Africa |  |
| Frank Plani  Suite 103  47 Clinton Rd New Redruth  Alberton, 1449 | 4 |
| Murimisi Mukansi  First Floor  1 Perth Road  Auckland Park, Gauteng, 2006 | 4 |
| Francois Erasmus  Suite 206  511 Jochemus street  Erasmuskloof X3 Pretoria, Gauteng, 0048 | 1 |
| Anwar Hoosen/Thea vanderHeever  1 Logeman Street  Bloemfontein, 9301 | 2 |
| South Korea |  |
| Sang Hak Lee  180 Wangsan ro Dongdaemun gu  Seoul, 02559 | 1 |
| Sang-Bum Hong  86 Asanbyeongwon-gil, Songpa-gu  Seoul, 05505 | 2 |
| Seok Chan Kim  222 Banpo-daero. Seocho-Gu  Seoul, 06591 | 4 |
| Spain |  |
| Eugenio Herrero,  Ctra. de Torrevieja a San Miguel de Salinas CV 95 Pda la Cenuela S/N  Servicio de Farmacia  Torrevieja, 03186 | 2 |
| Maria de la Cruz Martin  C/ Mateo Inurria, s/n  Servicio de Medicina Intensiva Planta 2  Torrejon de Ardoz, 28850 | 3 |
| Ricard Ferrer/Josep Trenado Alvarez  Pl Dr Robert 5  Servicio de Medicina Intensiva Planta 4 UCI Semicriticos  Tarrassa, 08221 Spain | 2 |
| Sri Lanka |  |
| Vasanthi Pinto  Professorial Medical Unit, Department of Medicine, Ward 17  Teaching Hospital  Peradeniya, 20400 | 1 |
| Taiwan |  |
| Ming-Cheng Chan  No. 1650 Sec 4 Taiwan Boulevard  Xitun Dist. Room 314 3F Research Building  Taichung, 40705 | 3 |
| Chih-Yen Tu  No. 2 Yu der Road  Taichung, 40447 | 2 |
| Yu-Feng Wei  No. 1, Yida Rd., Jiaosu Village  Yanchao District Kaohsiung, 82445 | 1 |
| Thailand |  |
| Chaicharn Pothirat  Div. of Pulm. Critical Medicine  2nd Fl., Sujinno Bldg.  Chiang Mai, 50200 | 4 |
| Methee Chayakulkeeree  2 Prannok Road  Bangkoknoi Bangkok, 10700 | 1 |
| Turkey |  |
| Serhat Unal  Hacettepe UTF Enfeksiyon Hastaliklari ABD Sihhiye  Ankara, 06100 | 3 |
| Iftihar Koksal  Karadeniz Teknik UTF Farabi Hastanesi Enfeksiyon Hastalıklari - Kalkinma Cad. Trabzon, 61080 | 3 |
| Ukraine |  |
| Vadym Nikonov  3A, Balakireva Line  Kharkiv, 61018 | 1 |
| Ivan Titov  91 Fedkovycha Str.  Ivano-Frankivsk, 76008 | 21 |
| Oleksandr Pavlov  1 Balakireva entry  Kharkiv, 61018 | 22 |
| Igor Kaydashev  27 a, Engelsa Str  Therapeutic Department, Chair of Internal Medicine #3  Poltava, 36038 | 2 |
| Jaroslav Pidhirnyj  7 Chernihivska Str  Lviv, 79010 | 2 |
| USA |  |
| Martin A. Croce  910 Madison Ave, 2nd Floor  Memphis, TN, 38163 | 10 |
| Lee E. Morrow  7710 Mercy Road, Suite 228  Omaha, NE, 68134 | 3 |
| Christopher S. Nelson/Jacob Quick  One Hospital Drive  Columbia, MO, 65201 | 5 |
| Paula Peyrani  501 E. Broadway  Suite 140  Louisville, KY, 40202 | 1 |
| Firas A. Koura  200 Medical Center Drive  Suite 2M  Hazard, KY, 41701 | 1 |
| Vidya Sundareshan  751 North Rutledge Street  Springfield, IL, 62702 | 1 |

**Supplementary** Table S7. Reasons for Exclusion From the CE and mITT Populations^a^

| Population | Tedizolid | Linezolid |
| --- | --- | --- |
| CE | **Not included in the CE population (n=90)**  No protocol-specified diagnosis of vHABP/VABP (n=10)  No gram-positive vHABP/VABP (n=9)  Received prohibited prior antibacterial therapy (n=13)  Received confounding concomitant antibacterial therapy (n=53)  Received incorrect study drug (n=0)  Study personnel unblinded during study (n=1)  Did not receive minimum dose amount (n=2)  Did not have clinical outcome assessment at TOC visit (n=13)  TOC visit occurred outside of allowable window (n=3)  Baseline or intercurrent medical events (n=4)  Unverified doses per pharmacy records (n=0) | **Not included in the CE population (n=118)**  No protocol-specified diagnosis of vHABP/VABP (n=8)  No gram-positive vHABP/VABP (n=13)  Received prohibited prior antibacterial therapy (n=14)  Received confounding concomitant antibacterial therapy (n=66)  Received incorrect study drug (n=4)  Study personnel unblinded during study (n=1)  Did not receive minimum dose amount (n=2)  Did not have clinical outcome assessment at TOC visit (n=17)  TOC visit occurred outside of allowable window (n=5)  Baseline or intercurrent medical events (n=6)  Unverified doses per pharmacy records (n=1) |
| mITT | **Not included in the mITT population (n=179)**  No acceptable respiratory specimen obtained (n=1)  Acceptable specimen obtained but  No growth of isolate (n=32)  Growth, but no gram-positive pathogen (n=146) | **Not included in the mITT population (n=159)**  No acceptable respiratory specimen obtained (n=2)  Acceptable specimen obtained but  No growth of isolate (n=15)  Growth, but no gram-positive pathogen (n=142) |

Abbreviations: CE, clinically evaluable; mITT, microbiological intention-to-treat; TOC, test of cure; VABP, ventilator-associated bacterial pneumonia; vHABP, ventilated hospital-acquired bacterial pneumonia.

^a^All populations were based on the actual treatment received. Reasons for exclusion were not mutually exclusive; a patient may have been excluded from a population for multiple reasons.

Supplementary Figure Legends
Supplementary Figure S1**.** Study design. Abbreviations: EOT, end of treatment; IV, intravenously; LFU, late follow-up; TOC, test of cure. ^a^Administered for 14 days if concurrent gram-positive bacteremia.

**Supplementary Figure S2.** Time to all-cause mortality (ACM) in the intention-to-treat population. Abbreviations: CI, confidence interval; NA, not available.

**Supplementary Figure S3.** Time to clinical failure in the intention-to-treat population.

**Supplementary Figure S4.** Investigator-assessed clinical response by patient subgroups.

Figure S1**.** Study design.


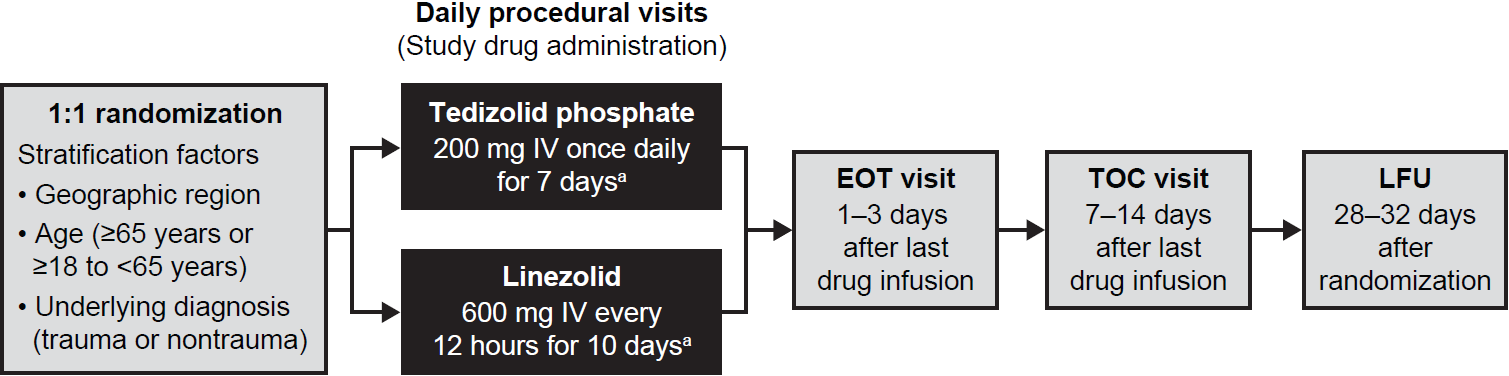


Abbreviations: EOT, end of treatment; IV, intravenously; LFU, late follow-up; TOC, test of cure.

^a^Administered for 14 days if concurrent gram-positive bacteremia.

**Figure S2.** Time to death in the intention-to-treat population.


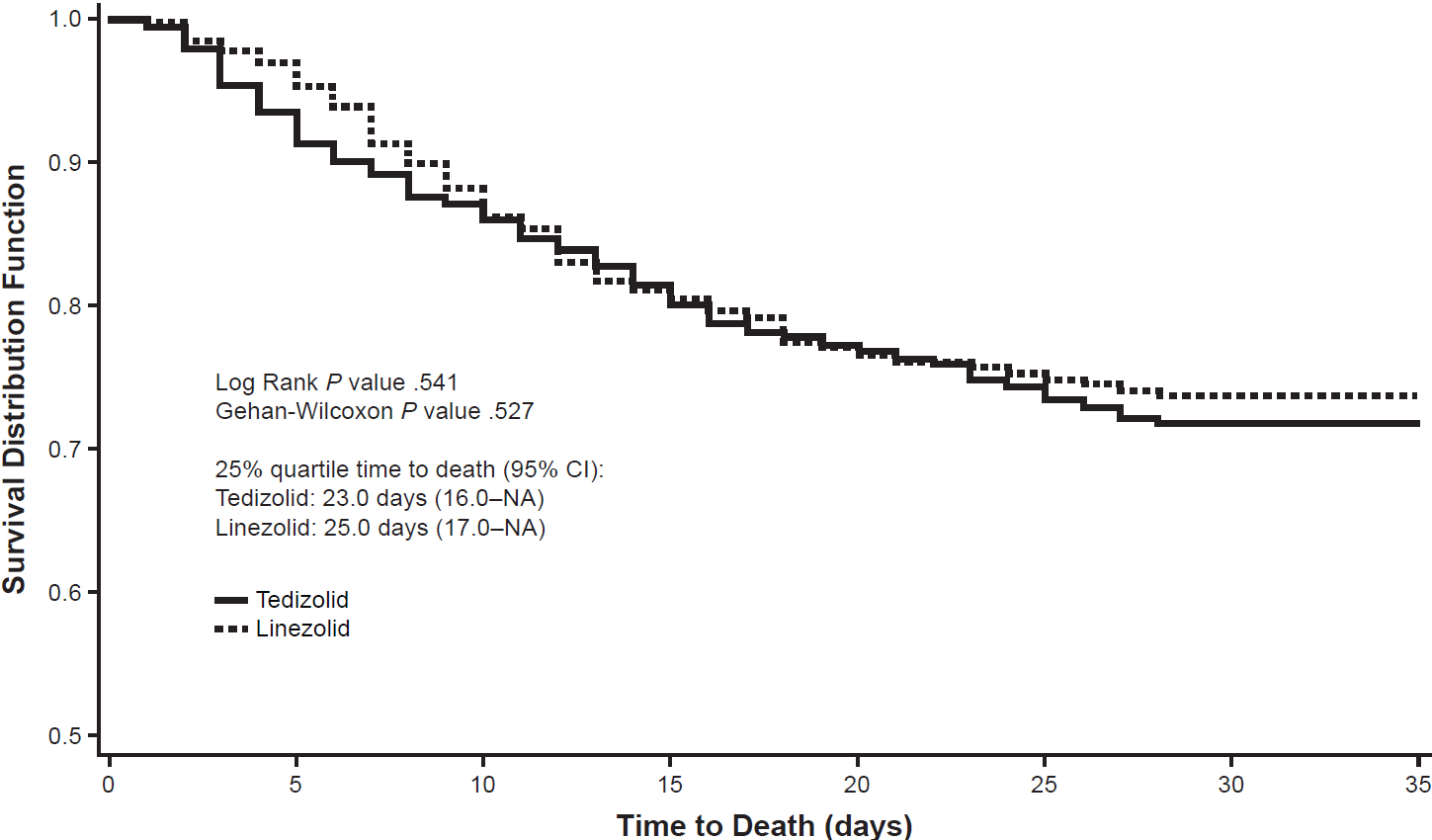


Abbreviations: CI, confidence interval; NA, not available.

**Figure S3.** Time to clinical failure in the intention-to-treat population.


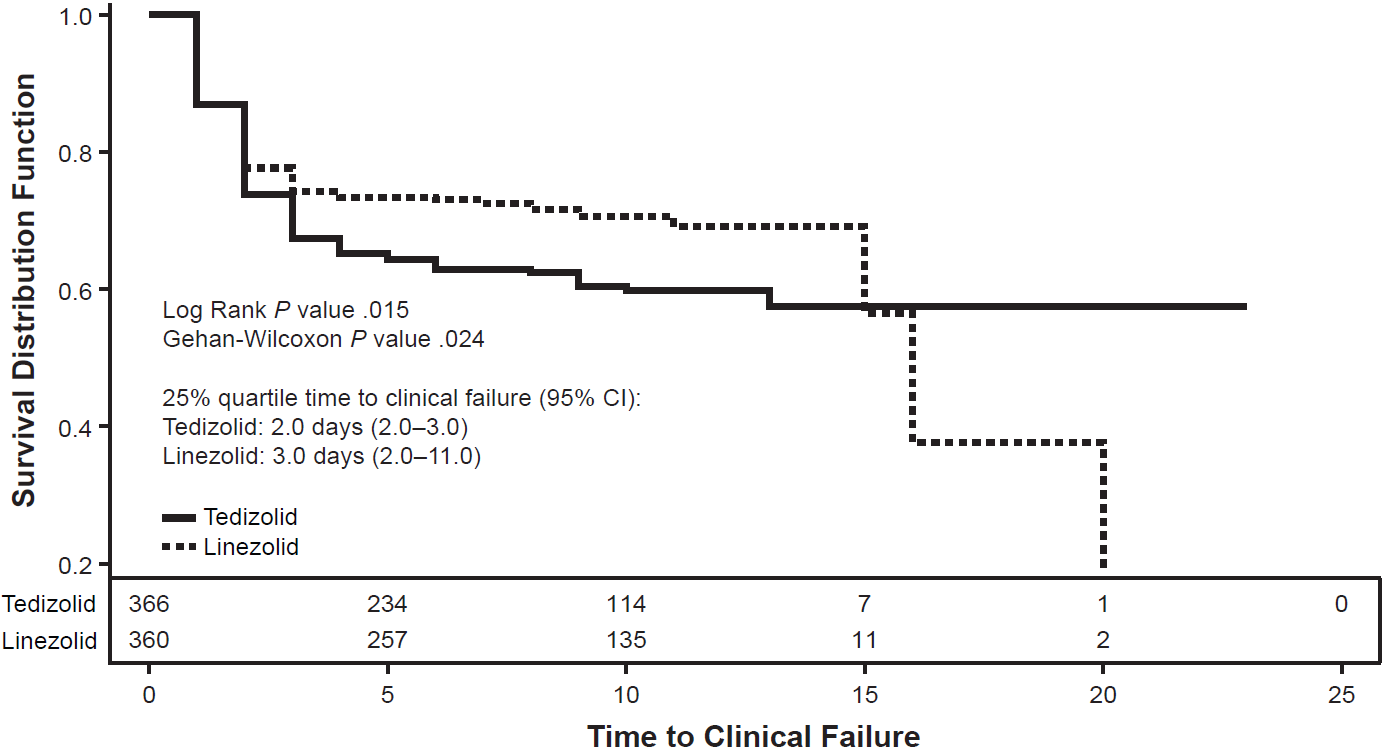


Abbreviation: CI, confidence interval.

**Figure S4.** Investigator-assessed clinical response by patient subgroups.


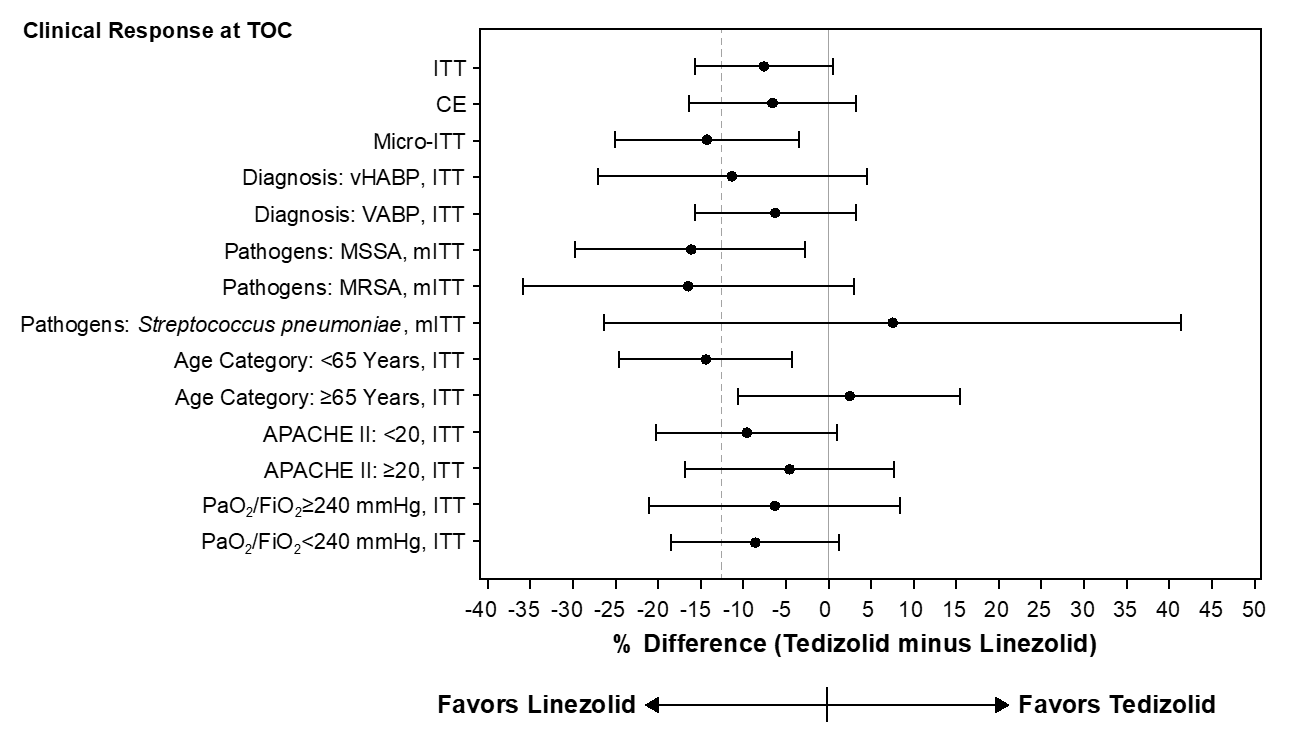


Abbreviations: APACHE, Acute Physiology and Chronic Health Evaluation; CE, clinically evaluable; FiO_2_, fraction of inspired oxygen; ITT, intention-to-treat; mITT, microbiological intention-to-treat; MRSA, methicillin-resistant *Staphylococcus aureus*; MSSA, methicillin-susceptible *Staphylococcus aureus*; PaO_2_, partial pressure of oxygen; TOC, test of cure; VABP, ventilator-associated bacterial pneumonia; vHABP, ventilated hospital-acquired bacterial pneumonia.
